# Case of Conical Cornea, Communicated

**Published:** 1830

**Authors:** James M. Staughton

**Affiliations:** Late Professor of Surgery in the Medical Department of the Columbian College, now of Cincinnati


Art. IV.—*Case of Conical Cornea, Communicated by* James M. Staughton, M. D., Late Professor of Surgery in the Medical Department of the Columbian College, now of Cincinnati.

In the summer of 1828, a young woman of 19 years of age, called at my office in Washington city, for advice. By the manner in which she groped her way into the room, I perceived that she was nearly blind. She was from the country, but was then on a visit to her sister at the Navy Yard. She complained of a dimness of vision in both eyes, which had been gradually increasing for two years. On looking at her eyes, which at first glance appeared natural, I recognised a case of conical cornea. I was in a moment struck with the truth of Travers' beautiful description of the disease, which he tells us, causes the cornea "to exhibit a degree of tenuity and brilliancy which gives it the appearance of a pellucid fluid like a dew drop suspended."

The alteration in the shape of the cornea was great and consequently the vision very imperfect. The patient could tell on which side of the room the windows were situated, but, so refracted were the rays of light that she could distinguish neither their form nor number. She was unable to walk alone in the streets, and it was by her sisters' arm and guidance that she reached my office. Nor was her sight much more distinct when the axis of vision was shortened. The most close approximation of the object to the eye, assisted her vision but little. I had in my possession a lens of unusual concavity, made for an optical instrument; this however she found to be of no assistance, as indeed usually happens.*

She complained of a continual sensation of fulness in the eye balls, amounting at times to acute pain. There was not, so far as the most attentive scrutiny could detect, the slightest vestige of inflammation in any of the tissues of the eye.

This was the first case of conical cornea, that had ever presented itself to my observation. From the various authors that had touched on the subject, I understood it to be an absorption of the interlamellar texture of the cornea, by which it looses its natural tonic resistance to the contents of the globe. I was forcibly struck with the great extension that the cornea had undergone, for the cavity of the aqueous humour was capable of containing more than double the usual quantity of fluid.

I am not disposed to deny the power of this interlamellar absorption to produce the disease—though I believe we have no post mortem examinations to prove it. On the contrary I think it would be difficult to account for the conoidal figure assumed, without supposing that the cornea afforded less resistance at the centre than at the edges. It appeared to me easy to account for the symptoms which were presented, by supposing the vessels which supply the aqueous humour to be in a state of increased activity. The gradual manner in which the vari-

---

*The most extreme cases of near sightedness resemble the disease in question, in the projection, though not in the conoidal shape of the cornea, and many of the most prominent symptoms are very similar. A remarkable case of this kind occurred in a Baptist Clergyman, now no more, well known for his eccentricities, and much respected for his unaffected piety and benevolence, a resident of New-Jersey. He was so near sighted as to be unable to walk without guidance. He could not read without approaching the book within an inch of his eye and even then could not see a letter unless he were in the open air, and the sun shining strongly on the page. Notwithstanding these difficulties, he mastered the languages. In the Greek he was truly learned, and being a man of great natural shrewdness, some of his philological researches were deemed truly valuable. His ingenious friend the late Rev. Dr. Allison, ground concave glasses for his use in reading, but so great was the requisite concavity, that the size of the letters was diminished so as to be scarcely perceptible.

Of course if lenses are used in the disease before us, they must be ground into a hollow conoidal shape, and not concave.

ous tissues extend before a slowly accumulating secretion, leaves no difficulty in accounting for the change of shape which occurs. Of the extensibility of the cornea, Burgman gives a frightful instance in a man who was hanged, whose cornea were so prodigiously distended that they reached down to the mouth like horns. HALLER. DISPUTAT. CHIRURG. VOL. II.

That a continued pressure was kept up in the eyes, the painful sensations of the patient, most abundantly proved. If the alteration in the shape were to be attributed to the pressure of the contents of the globe on the attenuated cornea, then ought all those contents in a case of great extension, like the present to be carried greatly forward—then ought the iris to present a convexity on its anterior surface—then ought the cornea to be soft and yielding to the touch. Now, in the case before us, I could find no convexity of the iris, and when the cornea was felt beneath the lid, which it caused to project very much, I found it firm and resisting. From this view of the subject, I came to the conclusion, that the disease was owing to an increased accumulation of the aqueous humour, and I determined to try if I could not reduce its quantity by some of the means within our reach. How far this view was correct, the result of the case will prove.

One word more. Mr. Wardrop remarks, that in those cases which he had examined, there has always been a small portion of the cornea where the surface was irregular, having the appearance which is observed, where a thin membrane fills up a part of the cornea which has been previously destroyed by ulceration. Hence he concludes that conical cornea is always the result of a structural or organic change. In one case of Mr. W.'s there was perceived by the patient a multiplication of images, and there must have been inequalities on the surface of the cornea, not, perhaps, unlike the various facets which the lapidary gives to the crystal, but of much greater irregularity. This case his friend, the celebrated Dr. Brewster of Edinburgh, examined with great attention. The Doctor was led to the conclusion from this and several other similar cases, that an irregularity of the corneal surface must be present in all possible cases of conical cornea : and he proposed to relieve it by glasses ground so as to correct the irregular refraction.

Far be it from me to suppose that the irregular surface does not at times accompany conical cornea. Though I must be excused from believing that Dr. Brewster, or any other optician could grind glasses to suit the varieties of irregularly refracting cornea. This opinion, however, I think explains very satisfactorily the manner in which, what Mr. Travers calls "*the tubular spectacle frame with a pupillar aperture*" renders the vision of the patient in certain cases, more distinct than any other contrivance ; for then the rays of light can be made to fall on a small portion of the smooth and uninjured cornea, and thus undergo a regular, though excessive refraction, while the rays are broken up and dispersed when they fall on the uneven surface of the whole cornea.

Mine however, was not a case of this kind. The most minute examination after Dr. Brewsters' most approved method did not shew any irregularity on the surface of the cornea. Nor did a pin hole in a card (which might perhaps answer as well as the "*tubular spectacle frame, &c.*") render the vision one whit more distinct.

The known properties of the iodine in promoting absorption presented themselves to my mind as offering the best means to diminish gradually the quantity of the aqueous humour, and to produce a corresponding alteration in the shape of the cornea. I directed the patient to rub the surface of the lids with a small portion of the ointment of iodine, every night and morning.

The first application of the ointment generally vesicates. It did so in this case. My patient, however, courageously continued its employment, and in four days she had so far recovered her sight as to be able to walk alone from the Navy Yard to my dwelling, a distance of upwards of two miles. The convexity of the cornea I found much diminished, and its transparency in no way injured. She was in the highest spirits at so wonderful an improvement, and continued to attend with the greatest diligence to my directions. In less than three weeks, she was able to read and to sew, from which she had been debarred for many months. She was anxious to return to the country. I directed her to continue in the moderate use of the iodine.

From that period I never heard of her till April 1830, when she came into the City to procure another box of the ointment. Her vision had remained distinct, and for upwards of a year she had not used the iodine. She had lately taken a severe cold and feared her old disease was returning. I detained her in the City, till she had used a portion of the iodine, and till she was perfectly satisfied that the disease was absolutely under the controul of the remedy.

Conical cornea is a rare disease. Many surgeons of great practice have never seen a case. I know of no remedy that has been so successfully used in the disease, as the iodine in the case related. Should a more extended trial prove its efficacy, I shall feel great pleasure in having thus communicated it to my professional brethren.

